# Short-Term Rhizosphere Effect on Available Carbon Sources, Phenanthrene Degradation, and Active Microbiome in an Aged-Contaminated Industrial Soil

**DOI:** 10.3389/fmicb.2016.00092

**Published:** 2016-02-05

**Authors:** François Thomas, Aurélie Cébron

**Affiliations:** ^1^CNRS, LIEC UMR7360, Faculté des Sciences et TechnologiesVandoeuvre-lés-Nancy, France; ^2^Université de Lorraine, LIEC UMR7360, Faculté des Sciences et TechnologiesVandoeuvre-lés-Nancy, France

**Keywords:** rhizosphere, plant root exudates, DNA/RNA, PAH, bacterial diversity, ryegrass (Lolium)

## Abstract

Over the last decades, understanding of the effects of plants on soil microbiomes has greatly advanced. However, knowledge on the assembly of rhizospheric communities in aged-contaminated industrial soils is still limited, especially with regard to transcriptionally active microbiomes and their link to the quality or quantity of carbon sources. We compared the short-term (2–10 days) dynamics of bacterial communities and potential PAH-degrading bacteria in bare or ryegrass-planted aged-contaminated soil spiked with phenanthrene, put in relation with dissolved organic carbon (DOC) sources and polycyclic aromatic hydrocarbon (PAH) pollution. Both resident and active bacterial communities (analyzed from DNA and RNA, respectively) showed higher species richness and smaller dispersion between replicates in planted soils. Root development strongly favored the activity of *Pseudomonadales* within the first 2 days, and of members of *Actinobacteria, Caulobacterales, Rhizobiales*, and *Xanthomonadales* within 6–10 days. Plants slowed down the dissipation of phenanthrene, while root exudation provided a cocktail of labile substrates that might preferentially fuel microbial growth. Although the abundance of PAH-degrading genes increased in planted soil, their transcription level stayed similar to bare soil. In addition, network analysis revealed that plants induced an early shift in the identity of potential phenanthrene degraders, which might influence PAH dissipation on the long-term.

## Introduction

During the last decade, progress in “meta-omics” approaches has greatly improved the understanding of bacterial communities in the rhizosphere of various plant species, revealing the complexity of plant-microbes interactions and their importance for soil ecosystems (Reinhold-Hurek et al., 2015). Most studies focused on unpolluted soil (Trivedi et al., 2012; Chaparro et al., 2014; Mendes et al., 2014), providing a fundamental knowledge of how plants shape the soil microbiome in the absence of perturbation. Comparatively little is known about the identity, activity, and short-term temporal dynamics of rhizospheric assemblages in contaminated habitats, although this could have significant impact for soil remediation on the long-term. Polycyclic aromatic hydrocarbons (PAHs) are one of the most abundant anthropogenic hazardous molecules in the environment. They consist of two or more fused aromatic rings produced from the incomplete combustion of organic matter, for instance in coking plants or gas factories. Over the past century, industrial activities left large areas of aged PAH-contaminated soil, threatening both ecosystems functions and human health. The high persistence of PAHs and their toxic, mutagenic, and carcinogenic properties have motivated the search for efficient remediation strategies (Gan et al., 2009). Among these, rhizoremediation of organic pollutants is based on the beneficial effects of plants on the degradation by soil microorganisms (Kuiper et al., 2004). This positive rhizosphere effect has been linked to increases in microbial abundance and activity due to root exudates providing carbon input, as well as the secretion of surfactant molecules or aromatic compounds homologous to PAHs (reviewed in Martin et al., 2014). Plants can significantly promote PAH degradation compared to bare soils (Binet et al., 2000; Liu et al., 2013; Storey et al., 2014) and this effect can partially be mimicked by amending soil with root exudates (Miya and Firestone, 2001; Joner et al., 2002). However, contrasting tendencies have been reported in the literature, where plants (Günther et al., 1996; Corgié et al., 2003; Liste and Prutz, 2006; Rezek et al., 2008) or exudates (Cébron et al., 2011; Phillips et al., 2012) can have no effect or even inhibit PAH degradation. So far, few studies have attempted to link PAH degradation to the impact of plants on the quality or quantity of available carbon sources, as well as their effect on the diversity and community composition of the resident and active soil microbiome. Among plant species, grasses offer some advantages for evaluating the rhizosphere effect on organic pollutant degradation and bacterial communities. They possess a fibrous root system allowing intensive penetration of the soil together with large surface area (Aprill and Sims, 1990; Dzantor et al., 2000) and exude high quantities of soluble organic substrates (Lynch and Whipps, 1990). Notably, ryegrass (*Lolium perenne*) was found as the most effective for PAH rhizoremediation in aged-polluted soil among 18 plant species representing eight families (Olson et al., 2007).

Therefore, the aim of the present study was to investigate the development of ryegrass rhizospheric microbiome in aged PAH-contaminated soil and its short-term influence on pollutant biodegradation. We hypothesized that the selection for specific members of the resident soil bacterial assemblage and changes in abundance and/or transcriptional activity in planted soil would impact PAH degradation, using phenanthrene (PHE) as a model PAH. Here we analyzed rhizospheric soil directly adherent to roots, bulk soil from planted microcosms as well as bare soil from non-planted controls, and compared the short-term dynamics of dissolved organic carbon (DOC), pollution (PHE), resident and active bacterial communities based on DNA/RNA analyses, and potential PAH degraders.

## Materials and methods

### Microcosm set-up

**Figure 1:**
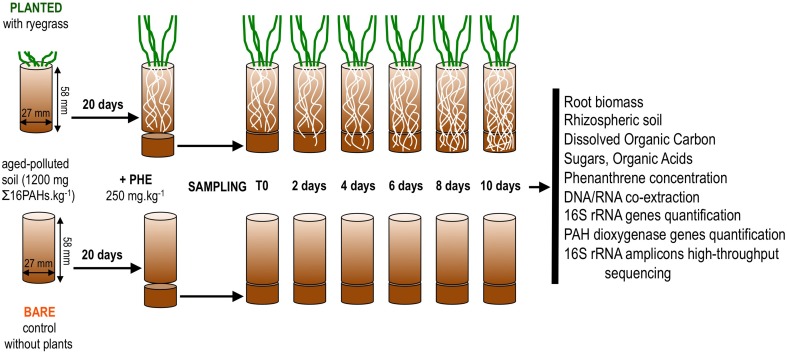
**Schematic summary of the microcosm set-up and subsequent sample analyses**.

Soil from a former coking plant site (NM soil, Neuves-Maisons, France; provided by the GISFI, www.gisfi.fr) was dried at room temperature, sieved to 2 mm and stored in the dark at room temperature until experimental set-up. Soil characteristics were detailed elsewhere (Cébron et al., 2009). This soil is mostly contaminated with aged PAHs (1260 mg Σ16PAHs.kg^−1^) and to a lesser extent with trace metals. To increase the bioavailability of pollutants, a batch of soil was spiked with phenanthrene (Fluka) at 250 mg.kg^−1^ as in Thomas et al. (2016). To allow contact of freshly spiked soil with growing roots, two-compartment microcosms were set up as shown in Figure 1. The first and second compartments consisted of the two parts of a sectioned 50 ml plastic syringe (internal diameter 27 mm; Codam Medicals ApS, DK). The first compartment (height 58 mm) was filled with 30 g of non-spiked soil wetted with 6.1 ml of sterile distilled water (80% of the soil water-holding capacity) and closed at the bottom with a nylon filtration mesh (porosity 500 μm). Eight ryegrass seeds (*Lolium multiflorum*, Italian ryegrass, Podium variety, LG seeds, France) were sown in triplicate first compartments and allowed to germinate at room temperature in the dark. After 3 days, they were transferred to the growth chamber (22/18°C day/night, 80% relative humidity, c.a. 250 μmol photons m^−2^ s^−1^, 16 h photoperiod) and the number of germinated seedlings was reduced to four to ensure similar numbers in all microcosms. Triplicate control first compartments without ryegrass were handled in the same way. Plants were grown until roots reached the bottom of the first compartment. After 20 days, a second compartment (height 15 mm, lined up with aluminum foil to minimize PAH adsorption to plastic and photodegradation) filled with 10 g of freshly PHE-spiked soil was attached below the first one. A disk of nylon filtration mesh (porosity 500 μm) separated the two compartments to prevent mixing of the two soils. Soil water content was adjusted to 80% of its water-holding capacity with sterile distilled water throughout the experiment, by weighing the microcosms. Water content did not differ between bare and planted microcosms throughout the experiment (average percent of water retention capacity: bare = 81 ± 2%; planted = 77 ± 6%; *t*-test *P* = 0.50). Triplicate microcosms were sacrificed for sampling after 2, 4, 6, 8, and 10 days, resulting in 15 bare soil samples and 15 planted samples. To capture the initial state of the soil, second compartments prepared in the same way were sacrificed within 15 min after rewetting (hereafter “day 0”). All sampling instruments were ethanol-sterilized and cleaned with RNaseZap (Life Technologies) before use. At the time of sampling, a scalpel blade was used to detach the first and second compartment below the nylon mesh disk. Soil from the second compartment was retrieved in a glass Petri dish. For ryegrass-planted microscosms, roots with adhering soil were gently sorted from bulk soil and transferred to another Petri dish. The adhering soil (hereafter “rhizospheric soil”) was carefully detached from roots using a brush and tweezers, whereas no further treatment was applied to bulk soil (hereafter “bulk planted soil”). The fresh weight of both soil fractions was measured on a digital balance (precision 10^−4^ g). Roots from the second compartment and shoots were oven-dried at 55°C for 2 days and weighed. Soil from non-planted microcosms (hereafter “bare soil”) was handled the same way. Soil samples were distributed as follows: 1 g in glass vial for PAH extraction; 0.5 g in plastic tubes for nucleic acid extraction (immediately frozen in liquid nitrogen); 6 g in plastic vials for DOC extraction. All samples were stored at −80°C until analysis. The sampling procedure took less than 30 min for each microcosm.

### Organic analyses

For PAH extraction and PHE measurement, soil was lyophilized and pulverized in a mixer mill (MM200, Retsch). Soil samples (250 mg) were mixed with diatomaceous earth (125 mg) and extracted with dichloromethane using an high pressure and temperature automated extractor Dionex ASE350 as described elsewhere (Biache et al., 2008). Solvent was exchanged to acetonitrile under nitrogen flux. PHE was quantified by reverse-phase chromatography using an HPLC System (Dionex) equipped with a Pursuit 3PAH column and a UV-Vis detector set at a wavelength of 254 nm, with a water:acetonitrile gradient.

For total DOC, carbohydrates and organic acids analyses, 6 g of soil were mixed with 30 ml of distilled water in Teflon tubes and agitated for 2 h at room temperature (Jones and Willett, 2006). Solutions were recovered by centrifugation at 3000 g for 10 min and filtered at 0.45 μm (polyester filter Chromafil PET-45/25, Macherey-Nagel). DOC was quantified on a TOC analyzer (TOC-V CSH, Shimadzu). Carbohydrates were measured by ion-exchange chromatography ICS 5000 equipped with a CarboPac SA10 column (Thermo Electron, Dionex) performed at the PTEF INRA-Nancy analytical platform. Sulfate ions were removed from samples prior to organic acid analysis to avoid column saturation. Briefly, aliquots (4 ml) were acidified to pH 2 with concentrated nitric acid, loaded on water-preconditioned Chromafix cartridges (PS-Ba2+, Macherey-Nagel), and neutralized with 0.2 M NaOH. Organic acids were measured as described elsewhere (Balland et al., 2010) using ion chromatography (ICS 3000, Dionex Corp.) equipped with an IonPac AS 11HC column (Dionex corp.), with conductivity detection.

### Nucleic acid extraction and cDNA synthesis

DNA and RNA were co-extracted from 0.5 g of soil using an adapted protocol from Wenderoth et al. (2003) with the Fast DNA Spin Kit for Soil (MP Biomedicals, France). Following recovery of DNA according to the manufacturer's instructions, RNA was precipitated from the suspension obtained after sedimentation of the binding matrix with 0.1 vol. 3 M sodium acetate pH 5.2 and 1 vol. isopropanol, overnight at 4°C. RNA was recovered by centrifugation 45 min at 19,500 g and 4°C, rinsed with 70% ice-cold ethanol, dried in a vacuum concentrator (Centrivap Jouan RC1010, ThermoScientific) and resuspended in RNase-free water (Gibco, Life Technologies). RNA samples were treated with 1 U of Turbo DNase (Ambion) and purified on RNeasy MinElute Clean-Up kit (Qiagen). Total elimination of DNA from RNA samples was checked by PCR of the 16S rRNA gene. DNA and RNA were quantified in 96-well plates using the Picogreen and Ribogreen assays (Invitrogen), respectively, following the manufacturer's instructions with standard curves from 0.03 to 0.5 ng.μl^−1^. Reverse transcription reactions were performed on 15 ng total RNA using the Superscript III First-Strand synthesis kit (Invitrogen) with random hexamers following the supplier's instructions. DNA and cDNA samples were diluted to 2 ng.μl^−1^ and 0.75 ng eq. RNA.μl^−1^, respectively, and stored at −20°C.

### Quantitative PCR

DNA and cDNA fractions were used to quantify bacteria using the primer pair 968F/1401R (Felske et al., 1998), targeting bacterial 16S rRNA genes. PAH-ring hydroxylating dioxygenase genes from *Proteobacteria* and *Actinobacteria* were quantified using the primer pairs PAH-RHDα GN F/R and PAH-RHDα GP F/R, respectively (Cébron et al., 2008). PAH-RHDα genes have been detected in both Gram-negative (GN) and Gram-positive (GP) bacteria. Within GN bacteria, genes have been identified in members of the *Alpha*-, *Beta*-, and *Gammaproteobacteria* and are specifically targeted by the PAH-RHDα GN F/R primer pair. *Actinobacteria* are the main Gram-positive soil bacteria involved in PAH degradation, and their PAH-RHDα genes is specifically targeted by the PAH-RHDα GP F/R. Although other bacteria might possess divergent genes that would not be targeted by this primer pair, the current lack of nucleotide sequence precludes the design of new primers. Quantitative PCR was performed as described in Cébron et al. (2008) on a CFX96 real-time system (BioRad). Reactions (20 μl) contained 1X iQ SybrGreen Super Mix (BioRad), 12 μg bovine serum albumin, 0.2 μl dimethyl sulfoxide, 40 ng of T4 bacteriophage gene 32 product (MP Biomedicals, France), 1 μl of template (DNA, cDNA, or 10-fold standard plasmid dilution series from 10^8^ to 10^2^ gene copies.μl^−1^) and 8 pmol of each primer. Reactions were heated at 95°C for 5 min, followed by 45 cycles of 30 s at 95°C, 30 s at the appropriate annealing temperature (56°C for 16S rRNA, 57°C for PAH-RHDα GN, 54°C for PAH-RHDα GP), 30 s at 72°C and 10 s at 82°C to capture the fluorescence signal while dissociating primer dimers. Dissociation curves were obtained by heating the PCR products from 50 to 95°C.

### PAH-RHDα genes clone libraries

Clone libraries were built with PAH-RHDα GN and GP amplicons obtained by qPCR on cDNA fractions from soil after 10 days incubation. qPCR reactions from triplicate microcosms were pooled. Amplicons were purified using the Cycle Pure Kit (Omega Bio-Tek) and cloned in the pGEM-T Vector System (Promega). Plasmids from ampicillin-resistant clones were purified using the E-Z 96 FastFilter Plasmid DNA kit (Omega Bio-Tek) and sequenced in one direction using the M13uni-21 forward primer. Sequences were edited in SeqTrace (Stucky, 2012) to clip PAH-RHDα primers and compared to the NCBI nr database using blastn (Madden, 2002) to retrieve closely related sequences. Alignments for PAH-RHDα GN and GP produced using MUSCLE (Edgar, 2004) were manually edited in MEGA6 (Tamura et al., 2013), and final versions comprising respectively 251 and 205 ungapped positions were used to infer maximum-likelihood phylogenetic trees with 100 resamplings.

### Illumina tag sequencing and analysis

The V3/V4 region of bacterial 16S rRNA genes was amplified using primers S-D-Bact-0341-a-S-17 (5′-CCTACGGGAGGCAGCAG-3′) and S-D-Bact-0787-b-A-20 (5′-GGACTACNVGGGTWTCTAAT-3′; Muyzer et al., 1993; Caporaso et al., 2010b) using a previously described dual-index paired-end strategy (Kozich et al., 2013). This primer pair has an overall coverage of 79% for Bacteria [tested on Silva TestPrime in July 2015, allowing 1 mismatch but no mismatch in the last four bases at 3′-end, (http://www.arb-silva.de/search/testprime/)]. PCR reactions were performed on 1 μl of template (DNA or cDNA) in a final volume of 20 μl, containing 1X Accuprime Super Mix (Invitrogen), 10 pmol of each primer (containing Illumina adapter and barcode), 12 μg bovine serum albumin, 0.2 μl dimethyl sulfoxide and 40 ng of T4 bacteriophage gene 32 product (MPBiomedicals, France). Reactions were heated at 95°C for 2 min, followed by 28 cycles of 20 s at 95°C, 15 s at 55°C, 5 min at 72°C, and a final extension step for 10 min at 72°C. Amplification products were checked by electrophoresis on 1% agarose gel and purified using the UltraClean-htp 96 Well PCR Clean-Up kit (MOBIO). After quantification by Picogreen assay (Invitrogen), a 10 nM equimolar pool of amplicons was prepared, purified using Nucleospin PCR Clean-Up kit (Macherey-Nagel) and sequenced on a single lane of Illumina Miseq PE250 at the Georgia Genomics Facility (Athens, GA, USA). Paired-end reads trimmed to a minimum QScore of 20 were joined using Pandaseq (Masella et al., 2012) with the following criteria: 400 bp < length < 450 bp and no ambiguous bases. Sequence data were analyzed using QIIME v1.9 (Caporaso et al., 2010a) and the R package phyloseq (Mcmurdie and Holmes, 2013). Chimeras were detected *de novo* using UCHIME (Edgar et al., 2011) and sequences were clustered in Operational Taxonomic Units (OTUs) at 97% similarity using USEARCH v6.1 (Edgar, 2010) implemented in the *pick_open_reference_otus.py* script in QIIME. Taxonomy was assigned using the RDP classifier (Wang et al., 2007) with the Greengenes database v13_8 (McDonald et al., 2012). OTUs affiliated to mitochondria and chloroplasts were removed for further analysis. Datasets were rarefied to the lowest number of sequences per sample (16,683 reads). Morisita-Horn distance was used for Principal Coordinates Analysis (PCoA), PERMANOVA (*adonis*), and *betadisp* tests implemented in vegan (Oksanen et al., 2013). Canonical Correspondance Analysis (CCA) was performed in XLStat 2012 software (Addinsoft) based on the distribution of abundant OTUs (>1% in at least one sample) in cDNA libraries. After checking of variance inflation factors and correlation between environmental variables, acetate (correlated to formate) as well as fructose, sucrose, and xylose (correlated to glucose) were not used for CCA calculation. The detection of bacterial orders with differential abundance was performed in STAMP (Parks et al., 2014). Network modeling was performed separately for bare and bulk planted soil, using data from cDNA libraries with only abundant OTUs (>1% in at least one sample) and environmental parameters. Local similarity analyses were performed using the eLSA software (Xia et al., 2011) with no delay. A total of 93 and 103 parameters were included in the LSA model for bare and bulk planted soil, respectively. LSA results were visualized using Cytoscape v3.1.1 (Shannon et al., 2003). Only associations with *P* < 0.01 based on 1000 permutations were considered significant (corresponding to *Q* < 0.005 and 0.012 for bare and bulk planted soil, respectively).

### Statistical analysis

Unless otherwise stated, statistical analyses were performed using R v3.1.3 (R. Core Team, 2013).

### Nucleotide sequence accession numbers

Sequence data for PAH-RHDα GN and GP clone libraries have been deposited in Genbank under accession numbers KT948521-KT948566 and KT948567-KT948613, respectively. Illumina MiSeq paired-end reads have been deposited in the SRA database under accession number SRP065058.

## Results

### Plant growth and soil carbon sources

**Figure 2:**
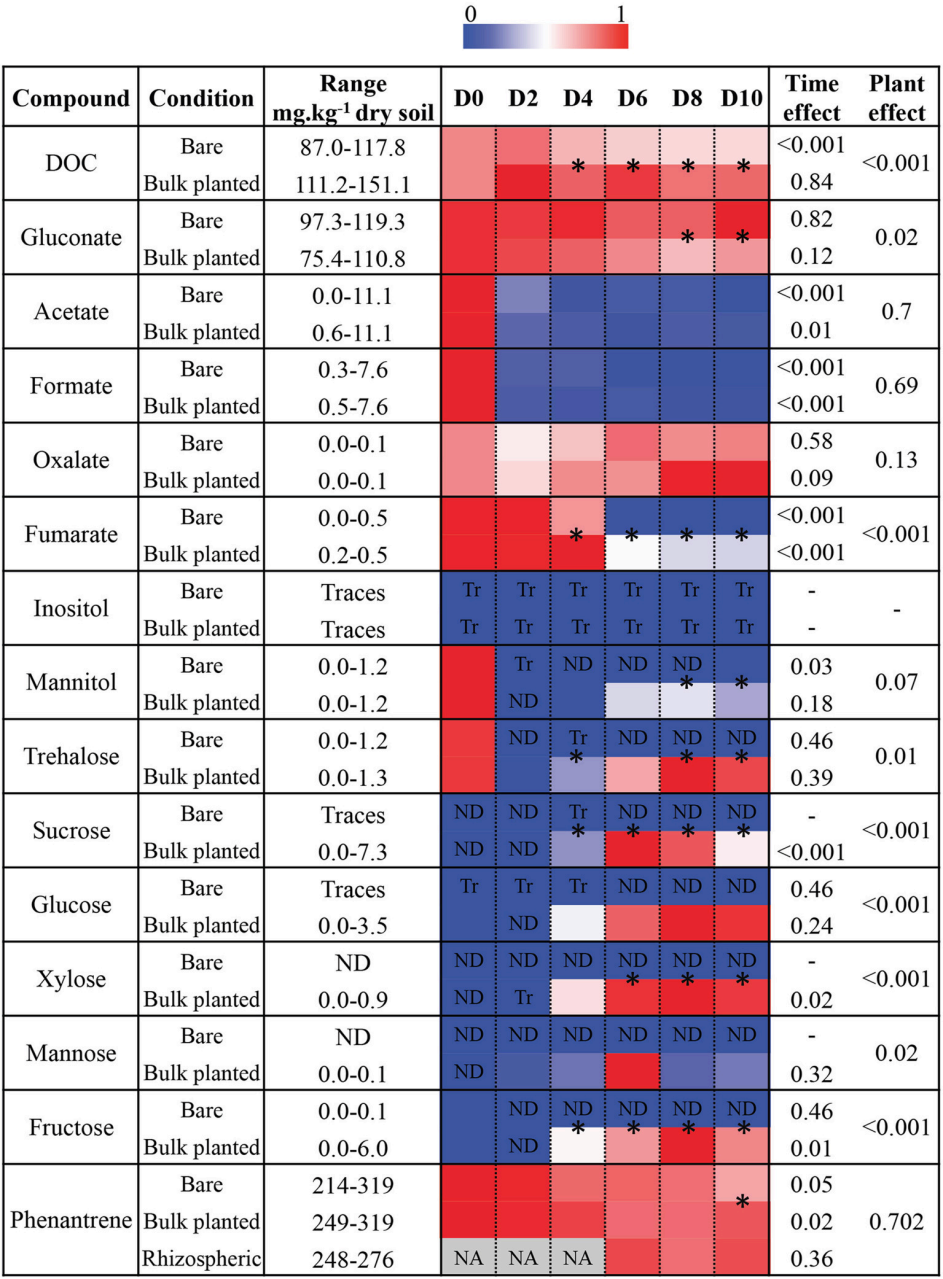
**Changes in total dissolved organic carbon (DOC), organic acids, sugars, and phenanthrene concentration in soil extracts over time, in bare and bulk planted soils from day 0 (D0) to day 10 (D10)**. For each compound, maximum detected concentrations were set to 1 and measurements were scaled accordingly. Values are means of three independent replicate microcosms. *P*-values obtained from one-way ANOVAs testing for the overall effect of vegetation and the effect of time for each condition are given. Asterisks between two adjacent cells denote significantly different values between bare and bulk planted soil (*t*-test, *P* < 0.05).

Following addition of the second compartment to the planted microcosms, ryegrass was actively growing as shown by the increase in aerial biomass, root biomass, and amount of rhizospheric soil over the 10-day kinetics (Figure S1). DOC significantly decreased over time in bare soil, likely due to microbial consumption (Figure 2). In contrast, DOC content stayed stable in bulk planted soil, due to the balance between carbon exudation by plants and microbial consumption. Analysis of soluble organic acids and sugars revealed that bulk planted soils contained significantly more fumarate, mannitol, trehalose, sucrose, glucose, xylose, mannose, and fructose than bare soils, where they were either absent or rapidly depleted. In contrast, the presence of plants tended to decrease gluconate content compared to bare soils. Acetate and formate were rapidly depleted in both conditions, and inositol was only detected in trace amounts.

The initial PHE concentration reached 319 ± 7 μg.g^−1^ dry weight soil after spiking with fresh PHE (Figure 2) compared to ca. 100 μg.g^−1^ in non-spiked soil. Phenanthrene contamination decreased significantly with time in both bare and bulk planted soil. PHE degradation rates reached respectively 9.9 ± 2.4, 6.5 ± 1.4, and 5.8 ± 1.8 μg.g^−1^.day^−1^ in bare, bulk planted and rhizospheric soil, revealing that plants slowed down PHE biodegradation. After 10 days, concentrations reached significantly lower levels in bare microcosms (214 ± 17 μg.g^−1^ dry soil, i.e., 48% degradation of spiked PHE) than in bulk planted (265 ± 8 μg.g^−1^ dry soil, i.e., 24%) and rhizospheric soil (272 ± 2 μg.g^−1^ dry soil, i.e., 21%).

### Abundance and transcriptional activity of total bacterial community and potential PAH-degraders

Bacterial 16S rRNA gene abundances were similar in bare and bulk planted soil throughout the 10-days time course (Table 1). At day 6, bacterial abundance was 2.3-fold higher in rhizospheric soil compared to bulk planted soil. The transcriptional activity of bacteria (16S rRNA transcripts) increased over one order of magnitude during the first 2 days, and was significantly higher in rhizospheric soil compared to bulk planted soil after 8 and 10 days.

**Table 1:** **Quantification of bacterial 16S rRNA and PAH–RHD_α_ genes and transcripts in soils from 0 to 10 days**.

		**Day 0**	**Day 2**	**Day 4**	**Day 6**	**Day 8**	**Day 10**	**Time effect**
***16S rRNA***								
DNA	Bare	2.1 ± 0.1 × 10^8^	4.2 ± 1.1 × 10^8^	6.8 ± 2.2 × 10^8^	6.4 ± 0.7 × 10^8^ (AB)	7.4 ± 0.7 × 10^8^	8.6 ± 3.3 × 10^8^	^*^
	Bulk planted	–	5.2 ± 0.8 × 10^8^	4.8 ± 0.4 × 10^8^	3.7 ± 0.8 × 10^8^ (B)	5.1 ± 0.9 × 10^8^	5.7 ± 0.7 × 10^8^	n.s.
	Rhizospheric	–	–	–	8.5 ± 1.2 × 10^8^ (A)	6.1 ± 0.5 × 10^8^	8.0 ± 0.6 × 10^8^	n.s.
cDNA	Bare	1.1 ± 0.1 × 10^8^	7.0 ± 4.3 × 10^8^	2.1 ± 0.1 × 10^8^	1.1 ± 0.1 × 10^9^	1.3 ± 0.3 × 10^9^ (AB)	1.0 ± 0.4 × 10^9^ (AB)	^**^
	Bulk planted	–	1.1 ± 0.3 × 10^9^	5.8 ± 1.5 × 10^8^	9.4 ± 1.2 × 10^8^	5.3 ± 0.2 × 10^8^ (B)	4.9 ± 1.0 × 10^8^ (B)	n.s.
	Rhizospheric	–	–	–	2.0 ± 0.6 × 10^9^	2.1 ± 0.8 × 10^9^(A)	2.2 ± 0.2 × 10^9^ (A)	n.s.
***PAH–RHD***_**α**_ ***GN (% ratio to 16S)***								
DNA	Bare	0.020 ± 0.003	0.131 ± 0.087 (B)	0.509 ± 0.261	0.099 ± 0.044 (B)	0.223 ± 0.046	0.036 ± 0.020 (B)	^*^
	Bulk planted	–	2.513 ± 1.107 (A)	1.637 ± 1.099	0.772 ± 0.251 (A)	0.498 ± 0.180	0.407 ± 0.165 (AB)	n.s.
	Rhizospheric	–	–	–	1.271 ± 0.270 (A)	0.821 ± 0.398	0.611 ± 0.30 (A)	n.s.
cDNA	Bare	b.q.	0.005 ± 0.005	0.003 ± 0.001	0.000 ± 0.000	0.001 ± 0.000	0.000 ± 0.000	n.s.
	Bulk planted	–	0.029 ± 0.014	0.008 ± 0.002	0.003 ± 0.001	0.001 ± 0.001	0.001 ± 0.000	^*^
	Rhizospheric	–	–	–	0.002 ± 0.001	0.001 ± 0.001	0.000 ± 0.000	n.s.
***PAH–RHD***_**α**_ ***GP (% ratio to 16S)***								
DNA	Bare	12.285 ± 0.950	12.603 ± 3.674	5.637 ± 1.763	6.594 ± 0.615	7.132 ± 0.138 (B)	4.902 ± 0.650 (B)	^*^
	Bulk planted	–	7.333 ± 0.134	8.378 ± 0.237	7.639 ± 0.546	11.032 ± 1.101 (A)	10.175 ± 0.887 (A)	^**^
	Rhizospheric	–	–	–	6.622 ± 0.432	11.044 ± 0.768 (A)	10.256 ± 0.373 (A)	^**^
cDNA	Bare	0.003 ± 0.003	0.009 ± 0.006	0.010 ± 0.005	0.144 ± 0.133	0.013 ± 0.007	0.012 ± 0.007	n.s.
	Bulk planted	–	0.009 ± 0.008	0.137 ± 0.111	0.033 ± 0.027	0.025 ± 0.006	0.118 ± 0.048	n.s.
	Rhizospheric	–	–	–	0.068 ± 0.057	0.032 ± 0.009	0.078 ± 0.045	n.s.

Values are mean ± s.e.m. (n = 3). b.q., below quantification limit of the qPCR assay. For statistical analysis, b.q. values were replaced by zero. Values for 16S rRNA and PAH-RHD were log- and arcsin-transformed, respectively. The effect of sample type (bare, bulk planted, or rhizospheric soil) was tested separately for each gene, time point, and nucleic acid using ANOVA with post-hoc Tukey test, and letters denote significantly different groups (P ≤ 0.05). The effect of time was tested separately for each sample type using ANOVA (

* P ≤ 0.05;

** *P ≤ 0.01)*.

Abundances of PAH-ring hydroxylating dioxygenase (PAH-RH_α_) genes from *Actinobacteria* (GP) were 1–2 orders of magnitude larger than PAH-RHD_α_ genes from *Proteobacteria* (GN) (Table 1). The percentage of PAH-RHD_α_ GN relative to 16S rRNA gene copies increased rapidly during the first 2 days, and this was more pronounced in bulk planted than bare soil. After 6 days, it started decreasing in bare microcosms but stayed stable and higher in bulk planted and rhizospheric samples. The percentage of PAH-RHD_α_ GP relative to 16S rRNA gene copies decreased over time in bare soil, whereas it increased to significantly higher levels in both bulk planted and rhizospheric samples. For both PAH-RHD_α_ GN and GP, no difference between bare, bulk planted, and rhizospheric soil could be detected in the number of transcripts relative to 16S rRNA throughout the 10 days time course. Cloning and sequencing of the amplicons retrieved from cDNA after 10 days showed that PAH-RHD_α_ GP clones were related to known sequences from *Mycobacterium* and *Arthrobacter* within *Actinobacteria* (Figure S2), whereas PAH-RHD_α_ GN clones clustered all with sequences from *Pseudomonas* (Figure S3).

### Dynamics of bacterial community

#### Overall bacterial community structure

**Figure 3:**
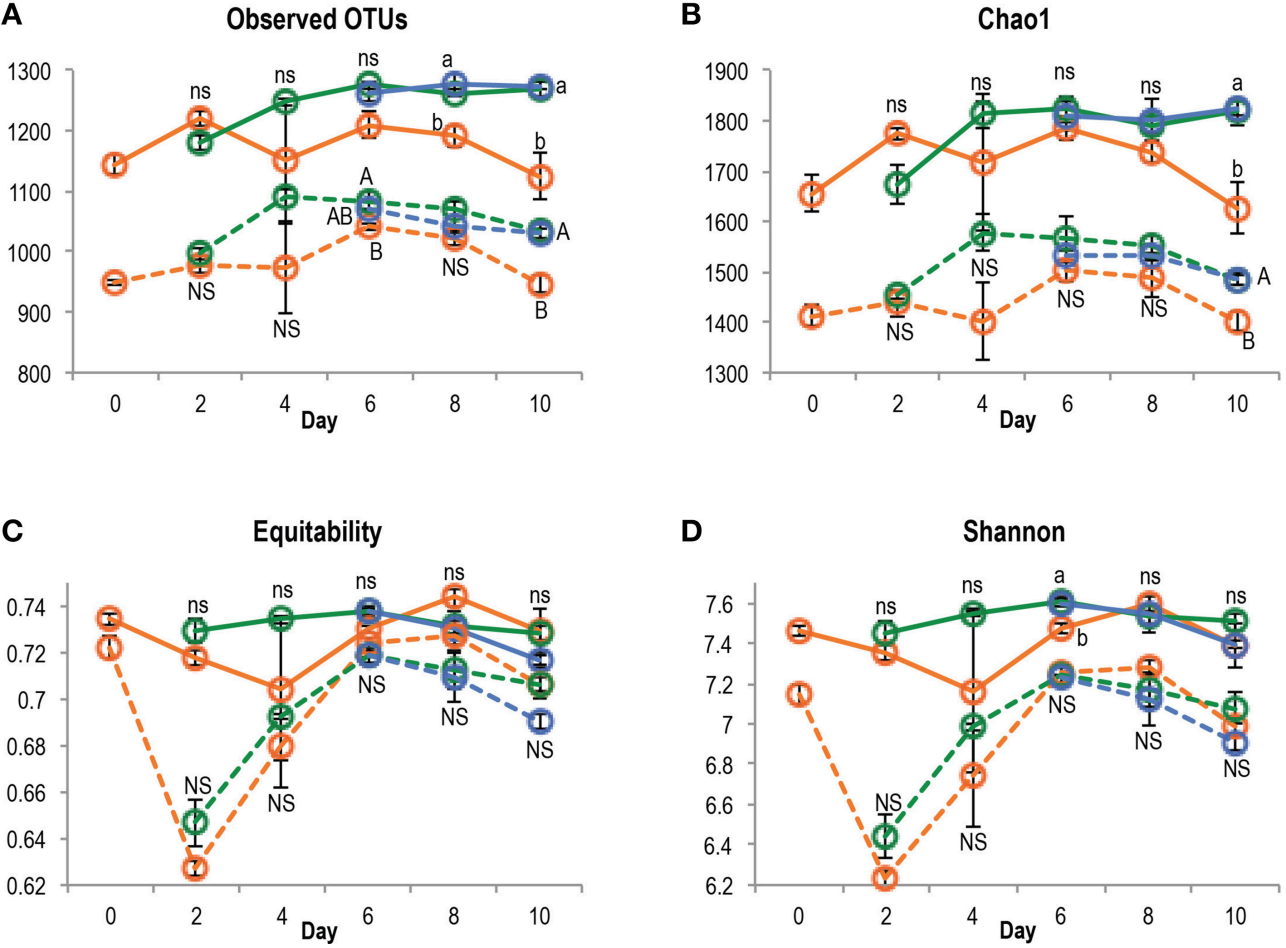
**Richness (A, number of OTUs; B, Chao1) and diversity (C, equitability; D, Shannon H′) indices in bare (orange), bulk planted (green), and rhizospheric soil (blue), based on DNA or cDNA libraries (plain and dotted line, respectively)**. The effect of sample type was tested separately for each time point and nucleic acid using ANOVA followed by *post-hoc* Tukey test, and letters denote group with significant difference (*P* < 0.05).

**Figure 4:**
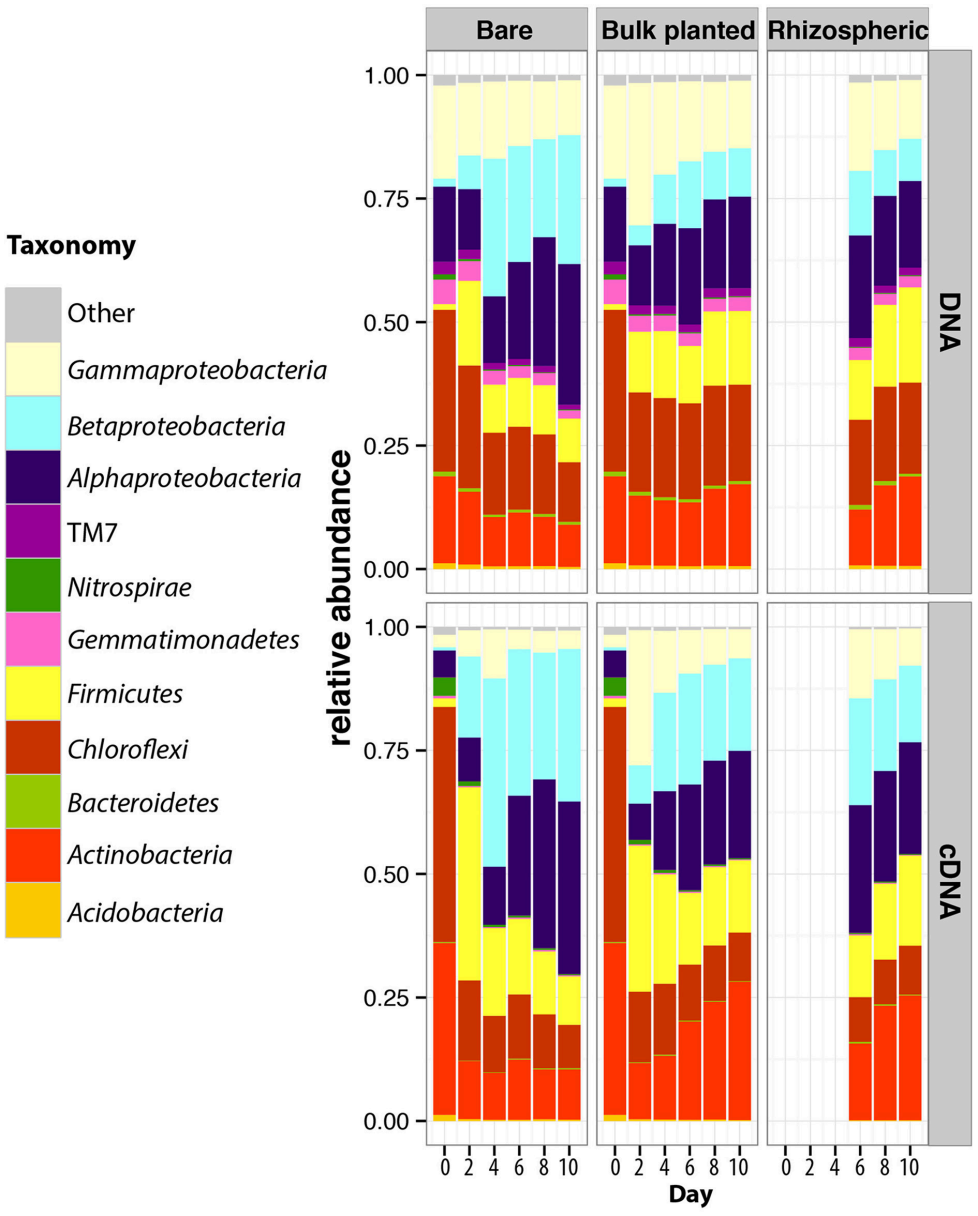
**Dynamics of the taxonomic composition of resident and active bacterial communities in bare, bulk planted and rhizospheric soil**. Values are mean of three independent microcosms. For comparison, data for day 0 in bare soil were also plotted for bulk planted soil.

After quality filtering, a total of 3,626,425 paired-end sequences were retrieved from the 84 DNA and cDNA 16S rRNA libraries, ranging from 16,683 to 106,000 reads per sample. Rarefaction analysis showed that the sequencing effort captured most of the diversity, with curves starting to reach a plateau (Figure S4) and Good's coverage above 97%. Richness (observed OTUs and Chao1) and diversity indices (Shannon and equitability) were significantly lower in cDNA libraries compared to DNA libraries (*t*-test, *P* < 0.001 for all indices), suggesting that only a subset of the total community was transcriptionally active (Figure 3). At both DNA and cDNA levels, richness was higher in bulk planted and rhizospheric samples compared to bare soil. Both resident and active communities were dominated by *Proteobacteria* (*Alpha*-, *Beta*-, and *Gammaproteobacteria* classes), *Chloroflexi, Firmicutes*, and *Actinobacteria* (Figure 4). Although *Acidobacteria, Bacteroidetes, Gemmatimonadetes*, and TM7 were detected in DNA libraries (range 0.4–4.0%), these phyla were not transcriptionally active (representation range in cDNA libraries: 0.01–0.39%). Correlations between abundances of OTUs in DNA vs. cDNA-based libraries were calculated to inform the contribution of resident bacterial populations to the active community. For all samples, number of reads at DNA and cDNA levels were positively correlated (Table S1, Spearman's ρ > 0.5), showing that in general activity was highly coupled to abundance for a specific OTU. No significant difference was found between the three types of samples at any time point. However, the correlation coefficient between rDNA and rRNA data increased significantly with time, suggesting that a larger proportion of the resident community became active along the 10-days incubation.

#### Effect of plant development on soil bacterial community

Principal Coordinates Analysis and Permutational Analysis of Variance (Figure S5) evidenced a major effect of the type of nucleic acid (DNA vs. cDNA) and samples (bare vs. bulk planted vs. rhizospheric) on the community composition, as well as an evolution with time. Furthermore, the dispersion of samples retrieved after 6–10 days was significantly higher in bare soil compared to bulk planted and rhizospheric soils, both at DNA (betadisp test, *F* = 9.4, *P* < 0.001; average distance to centroid: bare, 0.12; planted, 0.02; rhizospheric, 0.04) and cDNA level (*F* = 13.5, *P* < 0.001; average distance to centroid: bare, 0.17; planted, 0.03; rhizospheric, 0.05).

Based on data from cDNA libraries, a total of 12 active bacterial orders showed a significant difference in relative proportion, for at least one time point, between bare and bulk planted or rhizospheric soil (Table 2). Within the first 2 days, ryegrass root development strongly favored *Pseudomonadales* (seven-fold greater compared to bare soil) and decreased the contribution of *Bacillales, Sphingomonadales*, and *Burkholderiales*. Starting from day 4, *Caulobacterales* and *Rhizobiales* were stimulated by plants and this effect was more pronounced in rhizospheric soil compared to bulk planted soil. Within *Actinobacteria*, the contribution of *Acidimicrobiales* and *Actinomycetales* was higher in planted and rhizospheric samples compared to bare soil after 6 days, whereas *Solirubrobacterales* were specifically inhibited in rhizospheric soil. Finally, the proportion of *Rhodospirillales, Sphingomonadales*, and *Hydrogenophilales* was significantly lower after 10 days with ryegrass and in at least one prior time point.

**Table 2:** **Relative proportion of bacterial orders that significantly (*P* < 0.05) change with plant growth, based on data from cDNA libraries**.

	**Bare**	**Bulk planted**	**Rhizospheric**	***P***
**DAY 2**				
Firmicutes:Bacilli:Bacillales	**39.0 ± 1.3**	28.5 ± 2.3	-	0.019
Proteobacteria:Alphaproteobacteria:Sphingomonadales	**1.7 ± 0.0**	1.4 ± 0.1	-	0.032
Proteobacteria:Betaproteobacteria:Burkholderiales	**16.1 ± 1.8**	6.3 ± 1.8	-	0.023
Proteobacteria:Gammaproteobacteria:Pseudomonadales	3.7 ± 0.4	**26.8 ± 3.6**	-	0.006
**DAY 4**				
Proteobacteria:Alphaproteobacteria:Caulobacterales	4.4 ± 0.8	**7.5 ± 0.8**	-	0.050
Proteobacteria:Alphaproteobacteria:Rhizobiales	1.6 ± 0.1	**2.5 ± 0.2**	-	0.021
**DAY 6**				
Actinobacteria:Acidimicrobiia:Acidimicrobiales	5.4 ± 0.3 [B]	**10.4 ± 0.3 [A]**	6.5 ± 0.3 [B]	0.000
Actinobacteria:Actinobacteria:Actinomycetales	4.9 ± 0.1 [B]	**8.2 ± 0.6 [A]**	**8.1 ± 0.1 [A]**	0.002
Actinobacteria:Thermoleophilia:Solirubrobacterales	**1.1 ± 0.0 [A]**	**1.2 ± 0.1 [A]**	0.7 ± 0.0 [B]	0.001
Proteobacteria:Alphaproteobacteria:Caulobacterales	8.6 ± 0.4 [AB]	8.1 ± 0.7 [B]	**11.5 ± 0.6 [A]**	0.026
Proteobacteria:Alphaproteobacteria:Rhizobiales	2.4 ± 0.2 [B]	2.6 ± 0.1 [B]	**4.2 ± 0.3 [A]**	0.005
Proteobacteria:Betaproteobacteria:Hydrogenophilales	**9.8 ± 0.9 [A]**	6.2 ± 0.4 [B]	4.7 ± 0.5 [B]	0.009
Proteobacteria:Gammaproteobacteria:Pseudomonadales	1.6 ± 0.3 [B]	5.1 ± 1.0 [AB]	**8.7 ± 0.8 [A]**	0.004
Proteobacteria:Gammaproteobacteria:Xanthomonadales	1.6 ± 0.1 [B]	**2.5 ± 0.1 [A]**	**2.2 ± 0.1 [A]**	0.004
**DAY 8**				
Actinobacteria:Acidimicrobiia:Acidimicrobiales	4.4 ± 0.1 [B]	**10.0 ± 0.1 [A]**	**8.5 ± 0.7 [A]**	0.001
Actinobacteria:Actinobacteria:Actinomycetales	4.4 ± 0.2 [B]	**12.7 ± 0.6 [A]**	**13.8 ± 0.7 [A]**	0.000
Actinobacteria:Thermoleophilia:Solirubrobacterales	**0.9 ± 0.1[A]**	**1.0 ± 0.1 [A]**	0.6 ± 0.0 [B]	0.011
Proteobacteria:Alphaproteobacteria:Rhizobiales	2.1 ± 0.1 [B]	2.9 ± 0.1 [AB]	**4.5 ± 0.5 [A]**	0.012
Proteobacteria:Alphaproteobacteria:Rhodospirillales	**3.1 ± 0.5 [A]**	1.1 ± 0.1 [B]	1.0 ± 0.1 [B]	0.013
Proteobacteria:Alphaproteobacteria:Sphingomonadales	**20.6 ± 1.4 [A]**	7.6 ± 1.0 [B]	6.0 ± 0.8 [B]	0.000
**DAY 10**				
Actinobacteria:Acidimicrobiia:Acidimicrobiales	3.9 ± 0.2 [C]	**12.0 ± 0.2 [A]**	8.9 ± 0.2 [B]	0.000
Actinobacteria:Actinobacteria:Actinomycetales	4.9 ± 0.9 [B]	**15.0 ± 0.2 [A]**	**15.6 ± 1.3 [A]**	0.001
Firmicutes:Bacilli:Bacillales	10.2 ± 1.9 [B]	14.4 ± 0.8 [AB]	**18.2 ± 0.4 [A]**	0.025
Proteobacteria:Alphaproteobacteria:Caulobacterales	6.4 ± 0.1 [C]	9.5 ± 0.3 [B]	**12.4 ± 0.4 [A]**	0.000
Proteobacteria:Alphaproteobacteria:Rhizobiales	1.6 ± 0.2 [B]	**2.8 ± 0.2 [A]**	**3.5 ± 0.1 [A]**	0.001
Proteobacteria:Alphaproteobacteria:Rhodospirillales	**2.5 ± 0.3 [A]**	1.0 ± 0.0 [B]	0.8 ± 0.1 [B]	0.003
Proteobacteria:Alphaproteobacteria:Sphingomonadales	**23.3 ± 3.6 [A]**	8.0 ± 0.2 [B]	5.8 ± 0.2 [B]	0.005
Proteobacteria:Betaproteobacteria:Hydrogenophilales	**5.0 ± 0.2 [A]**	3.5 ± 0.2 [B]	3.5 ± 0.1 [B]	0.004

*Values are mean ± s.e.m. (n = 3) and bold face denote higher levels. For days 2 and 4, values in bare and bulk planted soil were compared using Student t-test. For days 6, 8, and 10, the effect of sample type was tested using ANOVA, with post-hoc comparison using Tukey-Kramer test*.

#### Structure of OTU-level bacterial community in relation with environmental variables

**Figure 5:**
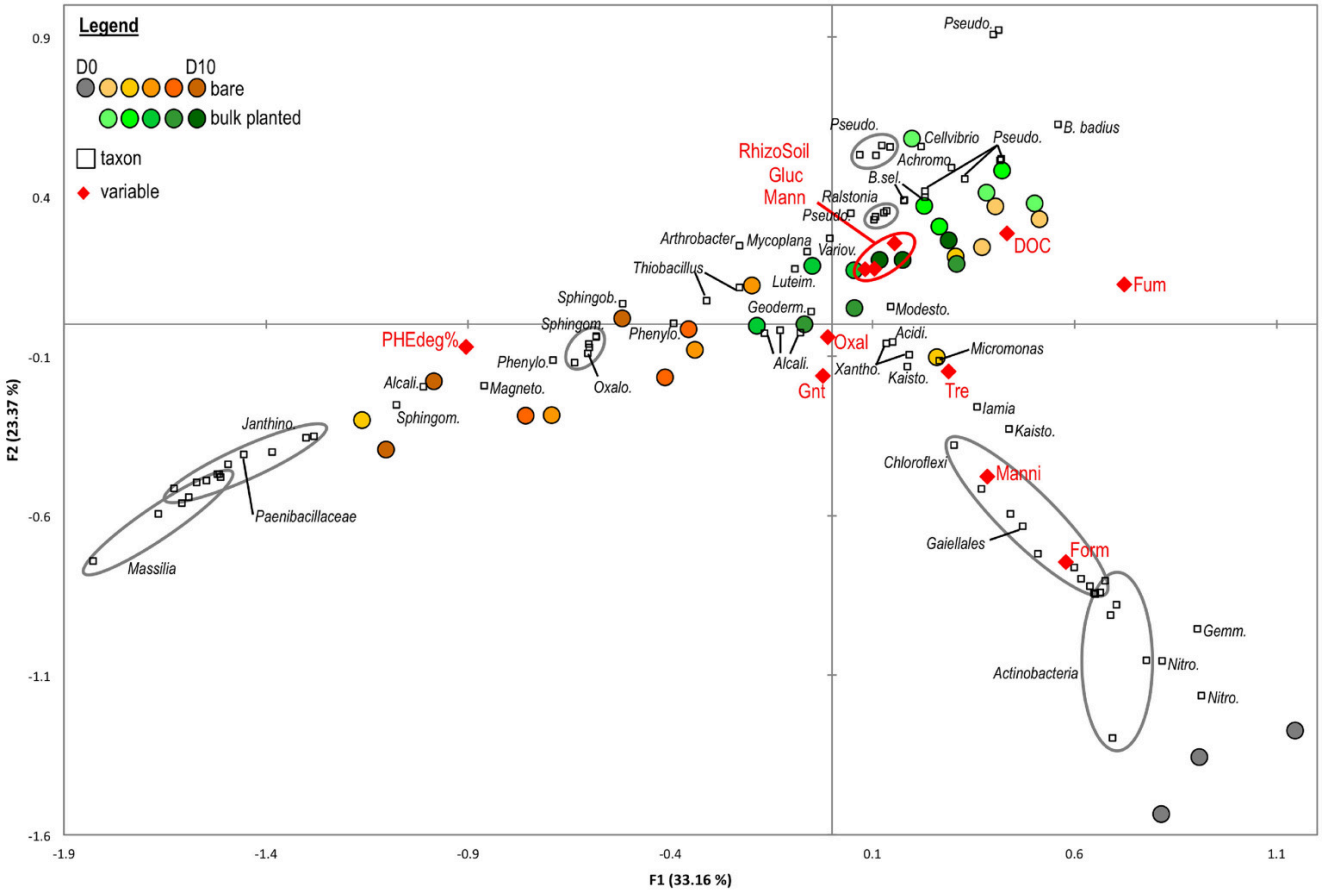
**Canonical Correspondance Analysis based on the distribution of abundant OTUs (>1% in at least one sample) in cDNA libraries**. The model accounted for 61.6% of the total inertia, with a global *P*-value < 0.0001. Circles represent samples from bare (green palette) or bulk planted soil (orange palette). Environmental variables (red diamonds) include percentage of PHE degradation (PHEdeg%), rhizospheric soil fresh weight (RhizoSoil), total dissolved organic carbon (DOC), glucose (Gluc), mannose (Mann), trehalose (Tre), mannitol (Manni), fumarate (Fum), oxalate (Oxal), gluconate (Gnt), and formate (Form). Squares represent OTUs with their taxonomic affiliation, abbreviated as follows: *Janthino*., *Janthinobacterium*; *Sphingom*., *Sphingomonas*; *Alcali*., unclassified *Alcaligenaceae*; *Magneto*., *Magnetospirillum*; *Phenylo*., *Phenlyobacterium*; *Oxalo*., unclassified *Oxalobacteraceae*; *Sphingob*., *Sphingobium*; *Geoderm*., unclassified *Geodermatophilaceae*; *Luteim*., *Luteiomas*; *Variov*., *Variovorax*; *Pseudo*., *Pseudomonas*; *B. sel*., *Bacillus selenatarsenatis*; *Achromo., Achromobacter*; *B. badius, Bacillus badius*; *Modesto*., *Modestobacter*; *Acidi*., unclassified *Acidimicrobiales*; *Xantho*., unclassified *Xanthomonadaceae*; *Kaisto*., *Kaistobacter*; *Nitro*., *Nitrospira*; *Gemm*., *Gemmatimonadetes*. For clarity, only OTUs with contribution >1% to at least one axis were depicted.

Canonical Correspondence Analysis (CCA) was used to test the influence of environmental variables on the OTU-level community structure, based on abundant OTUs (>1% in at least one sample) in cDNA libraries (Figure 5). The first axis of the CCA was positively correlated to fumarate and DOC, and negatively correlated to phenanthrene degradation rate. It separated samples primarily according to the presence or absence of plants, and secondarily according to the sampling time. Spearman tests confirmed the negative relationship of phenanthrene degradation with DOC (ρ = −0.33, *P* = 0.006), fumarate (ρ = −0.71, *P* < 0.001), acetate (ρ = −0.52, *P* < 0.001) and formate (ρ = −0.60, *P* = 0.001). The second axis of the CCA was negatively correlated with formate, and positively correlated with DOC and amount of rhizospheric soil. It mainly separated initial samples (day 0) from all others. OTUs related to *Chloroflexi, Actinobacteria* (specifically from the *Acidimicrobiales* and *Thermoleophilia*), *Nitrospira*, and *Gemmatimonadetes* were found predominantly in the soil at day 0 and correlated positively with formate, mannitol, and trehalose. Several OTUs affiliated with *Sphingomonas, Sphingobium, Magnetospirillum*, and *Phenylobacterium* and *Alcaligenaceae* correlated with phenanthrene degradation and prevailed in bare soil from late time points (>6 days). OTUs from *Pseudomonas, Cellvibrio, Achromobacter*, and *Ralstonia* were associated with bulk planted soils and correlated with DOC, glucose, mannose, and the amount of rhizospheric soil. Members of *Mycoplana, Variovorax, Luteimonas*, and *Actinomycetales* were also associated with bulk planted soils but without strong correlation to measured environmental variables. CCA based on DNA libraries (Figure S6) showed a relatively similar separation of samples according to growth conditions and environmental variables, revealing that changes in the community structure were at least partly visible in the resident community.

**Figure 6:**
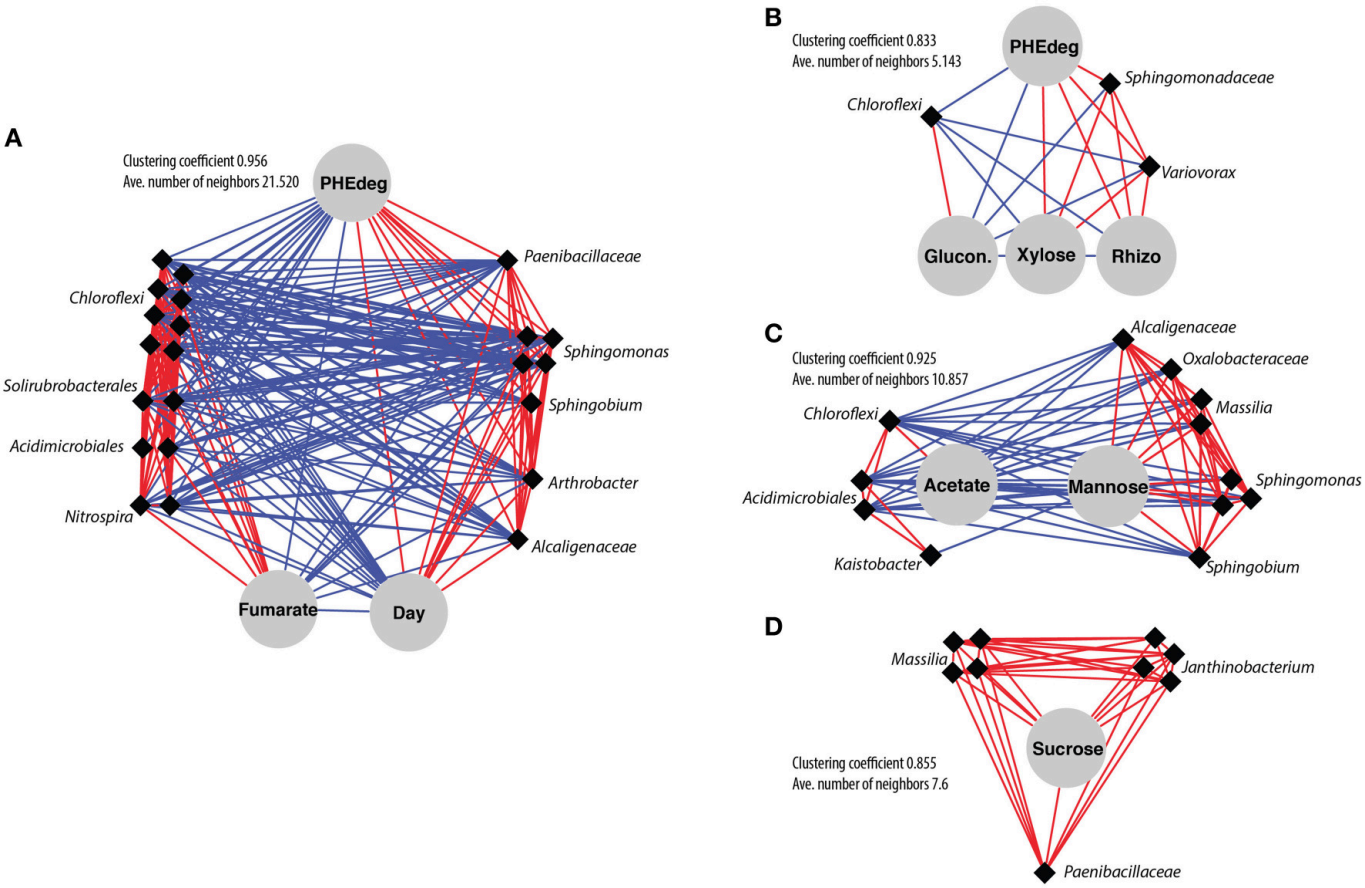
**Co-varying networks based on local similarity analysis of abundant OTUs in cDNA libraries (>1% in at least one sample) and environmental variables. (A)** Sub-network from bare soil centered around phenanthrene degradation. **(B–D)** Sub-networks from bulk planted soil centered around phenanthrene degradation **(B)**, mannose **(C)**, and sucrose **(D)**. Only associations with *P* < 0.01 were considered significant (*Q* < 0.005 and 0.012 for bare and bulk planted soil, respectively). Red and blue line edges represent positive and negative associations, respectively. OTUs are diamonds (with taxonomy) and environmental factors are circles.

Network analysis based on local similarity was used to reveal associations among abundant OTUs (>1% in at least one sample) and environmental parameters. Sub-networks organized around PHE degradation included 25 nodes when considering bare soil (Figure 6A) and only seven nodes for bulk planted soil (Figure 6B). Moreover, their degree of connectivity was higher in bare soil, as revealed by larger values of clustering coefficient and average number of neighbors. This suggests that the abundance of bacterial OTUs is more tightly coupled to the use of PHE as a carbon source in bare soil. Notably, PHE degradation was positively associated with a group of eight interconnected OTUs related to *Sphingomas, Sphingobium, Paenibacillaceae, Alcaligenaceae*, and *Arthrobacter* in bare soil, and only with 2 OTUs related to *Sphingomonadaceae* and *Variovorax* in bulk planted soil. By contrast, concentrations of plant-related labile compounds such as mannose (Figure 6C) and sucrose (Figure 6D) were highly associated with the abundance of OTUs related to *Sphingomonas, Sphingobium, Massilia*, and *Janthinobacterium* in bulk planted soil.

## Discussion

### Early development of rhizospheric bacterial communities in an aged-contaminated industrial soil

It is well established that the identity, abundance and activity of soil microorganisms depend on a variety of environmental parameters (e.g., soil type, temperature, pH, redox potential, etc.) as well as the presence of plants, their species and their development stage (Fierer and Jackson, 2006; Berg and Smalla, 2009; Reinhold-Hurek et al., 2015). The availability of organic matter is one of the most influential factors in the rhizosphere, since microbial growth in soil is generally carbon-limited (Wardle, 1992; Aldén et al., 2001). So far, knowledge on rhizodeposits derives mainly from systems where plants are grown in sand, inert materials (e.g., glass beads), or hydroponic cultures which might not reflect the quality and/or quantity of exudates released in natural conditions. Only few studies have analyzed rhizodeposits *in situ* from planted soils (Phillips et al., 2008; Neumann et al., 2009). In the present experiment, colonization of contaminated soil by actively growing roots provided new sources of organic substrates. The presence of plants was associated with higher concentrations of total DOC, as well as fumarate, mannitol, trehalose, sucrose, glucose, xylose, mannose, and fructose. This suggests that some of these molecules directly constitute part of the exudate cocktail released by ryegrass as shown previously in sand matrix (Clayton et al., 2008; Louvel et al., 2011). Moreover, microorganisms might release these simple compounds during the degradation of more complex exudates, breakdown of plant cell wall or as signals in response to the rhizospheric environmental conditions. Fungal species also produce carbohydrates such as trehalose (Elbein et al., 2003) and its detection might therefore be indirectly due to roots favoring fungal communities in planted soils (Thion et al., 2012). In contrast, concentration of other compounds such as acetate, formate, and inositol remained low in bulk planted soils, suggesting that they are either not released by ryegrass or that microbial consumption exceeds rhizodeposition. This modification of the soil carbon content quality and quantity in aged PAH-contaminated soil was shown for the first time to be accompanied by early shifts in the resident and active bacterial community structure, even if other plant-induced modifications (e.g., pH, redox conditions, etc.) could contribute. Species richness increased in planted microcosms, contrasting from what is commonly shown in non-contaminated soils where richness or diversity indices are usually lower in the presence of plants (Uroz et al., 2010; Li et al., 2014; Shi et al., 2015), including ryegrass (Marilley et al., 1998). Therefore, increase of bacterial richness as well as diversity in the rhizosphere might be characteristic of polluted soils, since it has already been shown for the same soil (Cébron et al., 2009; Bourceret et al., 2015) and other sites (Shahsavari et al., 2013; Li et al., 2015). This discrepancy could be due to the oligotrophic nature of contaminated soils, where nutrients are scarce and carbon sources are either too recalcitrant or not bio-available. In these extreme environments, plants can provide labile substrates and offer new microniches to favor the development of inactive bacterial species, leading to an increase in richness (Dejonghe et al., 2001). Such structuring of the soil in microniches was evidenced by the increase in transcriptional activity of bacteria, which was restricted to the root-adherent rhizospheric soil compared to bulk planted soil. In addition, plants reduced the variability in the community structure compared to bare soil, suggesting that although increasing spatial heterogeneity, the rhizosphere environment might provide more temporally stable conditions and constrain microbiome assemblage and activity.

The active community composition significantly changed as a result of root colonization. *Pseudomonadales* were favored early after root colonization (within 2 days) and could be considered as the first rhizosphere-responsive group, followed by *Caulobacterales* and *Rhizobiales* (4 days). More diverse communities were active after 6–10 days, notably characterized by members of *Acidimicrobiales, Actinomycetales, Caulobacterales, Rhizobiales, Pseudomonadales*, and *Xanthomonadales*. Many studies have previously detected these taxa in the rhizosphere of various plant species in unpolluted soils (DeAngelis et al., 2009; Turner et al., 2013; Chaparro et al., 2014; Li et al., 2014). Reversely, plants strongly selected against members of *Sphingomonadales* and to a lesser extent *Hydrogenophilales, Rhodospirillales, Comamonadaceae, Oxalobacteraceae*, and *Paenibacillaceae*. These shifts in bacterial community composition might be due to the increase in carbon availability via rhizodeposition favoring fast growing r-strategist bacteria (Fierer et al., 2007; Blagodatskaya et al., 2014). Furthermore, Goldfarb et al. (2011) showed a differential response of soil bacteria to substrates of varying chemical recalcitrance, some being favored with labile compounds (e.g., *Actinomycetales* and *Pseudomonadales*) and others with more recalcitrant carbon sources (e.g., members of *Sphingomonadales*). Interestingly, members of *Actinomycetales, Rhizobiales Pseudomonadales, and Xanthomonadales* were also favored in the same NM soil planted with alfalfa after 37 days in rhizotron experiments (Bourceret et al., in preparation) and after 6 years in a field trial (Bourceret et al., 2015), pointing to an early selection of a rhizospheric community that can persist throughout plant development and seasons.

### Early effects of plants on phenanthrene biodegradation

During the 10-days time course, the presence of ryegrass slowed down the degradation of spiked phenanthrene although the abundance of PAH RHD_α_ genes increased compared to bare soil. These effects were greatest in root-adherent rhizospheric soil. Rhizodeposits such as simple sugars and organic acids are more bioavailable and energetically favorable than PHE (Providenti et al., 1993; Woo and Rittmann, 2000) and may therefore be preferentially consumed by PAH-degrading bacteria, as revealed by the negative correlation between PHE degradation and DOC content. Although not specifically tested in this study, the reduction of PHE degradation in planted microcosms might also be partially linked to micronutrients depletion in the rhizosphere, possible adsorption of PAHs to roots or changes in pO_2_/pCO_2_ (Joner et al., 2002; Corgié et al., 2003). Previous investigations have proposed that plant-assisted organic pollutant remediation is a balance between the positive effect of rhizodeposits on microbial abundance vs. negative effect due to substrate competition (Kamath et al., 2004; Rentz et al., 2004; He et al., 2005; Fang et al., 2012). Therefore, the positive effect of plants on the abundance of PAH degraders reported here may on the long-term compensate for the competition with labile substrates or overcome inhibition as the quality and quantity of exudates evolve with ryegrass development. Interestingly, no difference in the number of transcripts of PAH-RHD_α_ could be detected between bare and planted soil throughout the kinetics, confirming that root colonization did not induce the expression of PAH-degrading pathways. The relatively low abundance of PAH-RHD transcripts may possibly indicate that the selected primers did not fully target the relevant degrading organisms. However, sequence data obtained from clone libraries confirmed that the amplified sequences were affiliated to *Pseudomonas, Arthrobacter*, and *Mycobacterium*, which are known as active PAH degraders in contaminated soils, including from Neuves-Maisons. Alternatively, the transcription might have increased very transiently before the first time point at day 2, or the stock of PAH-RHD enzymes in degrading bacteria might be sufficient to process the spiked PHE without an increase in the transcription. This lack of transcriptional induction also suggests that rhizodeposits did not trigger a massive carbon catabolite repression (CCR), which would expectedly yield lower transcription of PAH-RHD genes in planted soil. Purified sugars and root extracts have been shown to inhibit the degradation of aromatic compounds by CCR in several cultivated isolates (Keuth and Rehm, 1991; Dal et al., 2002; Kamath et al., 2004; Rentz et al., 2004; Rojo, 2010; Zhang and Anderson, 2013; Vandera et al., 2015). However, it has been suggested that CCR may be more significant when considering a single species of degrader rather than a diverse community (Martin et al., 2014). Furthermore, the present experimental set-up allowed testing the effect of low and continuous inputs of natural, complex rhizodeposits on autochthonous PAH degraders instead of high concentrations of purified compounds, which might explain that the limited impact of CCR was overlooked so far.

Plants also modified the identity of potential PAH degraders in the NM soil. In bare microcosms, major potential PHE utilizers included representatives of *Sphingomonas, Sphingobium, Phenylobacterium* and *Arthrobacter*, the abundance of which was positively correlated with PHE degradation rates. This is concordant with previous results on PHE-spiked agricultural soils, where *Sphingomonas* became the dominant genus within 2 months, together with a significant increase in *Phenylobacterium* abundance compared to unspiked controls (Ding et al., 2012). Sphingomonads are known as active hydrocarbon degraders (Peng et al., 2008), often isolated from polluted sites and able to use PHE and other PAHs as sole carbon sources (reviewed in Waigi et al., 2015). In general, sphingomonads feature several adaptive strategies for efficient PAH degradation in oligotrophic environments, such as: (i) adhesion to hydrophobic substrates and formation of biofilm (Johnsen and Karlson, 2004); (ii) the presence of glycosphingolipids in their cell envelope (Kawahara et al., 1999), which are more hydrophobic than common membrane lipids found in other Gram-negative bacteria; and (iii) high affinity uptake systems for recalcitrant compounds (Peng et al., 2008). Interestingly, the relative abundance of members of *Sphingomonadales* within the active community strongly decreased in planted samples and correlated more with the concentration of simple carbohydrates which have been shown to be used by cultivated *Sphingomonas* species (Balkwill et al., 1997; Denner et al., 2001). Due to their adaptation to oligotrophy, PAH-degrading sphingomonads might be rapidly outcompeted by other fast-growing soil bacteria in the carbon-rich rhizosphere. In contrast, potential PHE-degraders in planted soil include *Pseudomonas, Ralstonia*, and *Variovorax* representatives. Using the same NM soil for a stable isotope probing (SIP) experiment with ^13^C-PHE, Cébron et al. (2011) previously reported that the addition of ryegrass root exudates favored *Pseudomonas*, which dominated the ^13^C-labeled community. Recent isolation trials for PHE degraders from ryegrass-colonized NM soil retrieved nine new closely related *Pseudomonas* strains (Thomas et al., 2016), confirming their major role for PHE depollution in planted soils. Bacteria affiliated to *Variovorax* and *Ralstonia* were also identified as active PAH-degraders in contaminated soil, in SIP experiments using ^13^C-anthracene and naphthalene (Singleton et al., 2005; Jones et al., 2011a,b). In the future, SIP experiments using ^13^C-labeled PAHs coupled to metagenomics would give invaluable information to evaluate the impact of plant growth and rhizosphere development on the identity of active degraders as well as their metabolic pathways.

## Author contributions

Designed experiments: FT and AC. Performed experiments: FT. Analyzed results: FT and AC. Wrote the manuscript: FT and AC.

### Conflict of interest statement

The authors declare that the research was conducted in the absence of any commercial or financial relationships that could be construed as a potential conflict of interest.

## Supplementary material

Figure S1 **Evolution of root and aerial dry weight biomass, and rhizospheric soil fresh weight biomass in microcosms planted with ryegrass**. Values are mean ± s.e.m. (*n* = 3).

Figure S2 **Maximum-likelihood (ML) phylogenetic reconstruction of PAH-RHD GP nucleotidic sequences cloned from the Neuves-Maison soil (in boldface), including publicly available sequences from reference strains and uncultured bacteria**. The Tamura-Nei model was used for the ML analysis. Values of ML bootstrap support (100 resamplings) greater than 50% are reported. Unique sequences are represented by one selected clone; numbers of retrieved sequences from bare and bulk planted soil are shown in orange and green boxes, respectively. The tree was rooted on the *Rhodococcus/Arthrobacter* clade. The bar indicates 5% sequence divergence.

Figure S3 **Maximum-likelihood (ML) phylogenetic reconstruction of PAH-RHD GN nucleotidic sequences cloned from the Neuves-Maison soil (in boldface), including publicly available sequences from reference strains and uncultured bacteria**. The Tamura-Nei model was used for the ML analysis. Values of ML bootstrap support (100 resamplings) greater than 50% are reported. Unique sequences are represented by one selected clone; numbers of retrieved sequences from bare and bulk planted soil are shown in orange and green boxes, respectively. The tree was rooted on the *Polaromonas* branch. The bar indicates 2% sequence divergence.

Figure S4 **Rarefaction curves for OTUs clustered at 97% similarity**.

Figure S5 **Principal Coordinates Analysis (PCoA) displaying the Morisita-Horn dissimilarity between soil bacterial communities, based on DNA and cDNA libraries**. Data from bare, bulk planted and rhizospheric soil were analyzed all together but plotted on three superimposable panels for clarity. Permutational Analysis of Variance corroborated the effect of nucleic acid (DNA vs. cDNA; *F* = 43.0, *P* < 0.001, *R*^2^= 0.24) and type of samples (bare vs. bulk planted vs. rhizospheric; *F* = 11.8, *P* < 0.001, *R*^2^= 0.13) on the community composition, as well as an evolution with time (*F* = 33.7, *P* < 0.001, *R*^2^ = 0.19).

Figure S6 **Canonical Correspondance Analysis based on the distribution of abundant OTUs (>1% in at least one sample) in DNA libraries**. The model accounted for 59.9% of the total inertia, with a global *P*-value of 0.002. Circles represent samples from bare (green palette) or bulk planted soil (orange palette). Environmental variables (red diamonds) include percentage of PHE degradation (PHEdeg%), rhizospheric soil fresh weight (RhizoSoil), total dissolved organic carbon (DOC), glucose (Gluc), mannose (Mann), trehalose (Tre), mannitol (Manni), fumarate (Fum), oxalate (Oxal), gluconate (Gnt), and formate (Form). Squares represent OTUs with their taxonomic affiliation, abbreviated as follows: *Janthino*., *Janthinobacterium*; *Sphingom*., *Sphingomonas*; *Alcali*., unclassified *Alcaligenaceae*; *Phenylo*., *Phenlyobacterium*; *Oxalo*., unclassified *Oxalobacteraceae*; *Sphingob*., *Sphingobium*; *Pseudo*., *Pseudomonas*; *B. sel*., *Bacillus selenatarsenatis*; *Xantho*., unclassified *Xanthomonadaceae*. For clarity, only OTUs with contribution >1% to at least one axis were depicted.

Table S1 **Spearman correlation coefficient between DNA and cDNA read data**. Differences between conditions were tested using ANOVA followed by post-hoc Tukey-Kramer test. Letters denote groups with significant differences (*P* < 0.05).
