# Supplementary figures and images for: Short-Term Rhizosphere Effect on Available Carbon Sources, Phenanthrene Degradation, and Active Microbiome in an Aged-Contaminated Industrial Soil
Source: Front Microbiol. 2016 Feb 5;7:92. doi: 10.3389/fmicb.2016.00092 (PMC4742875; doi:10.3389/fmicb.2016.00092)

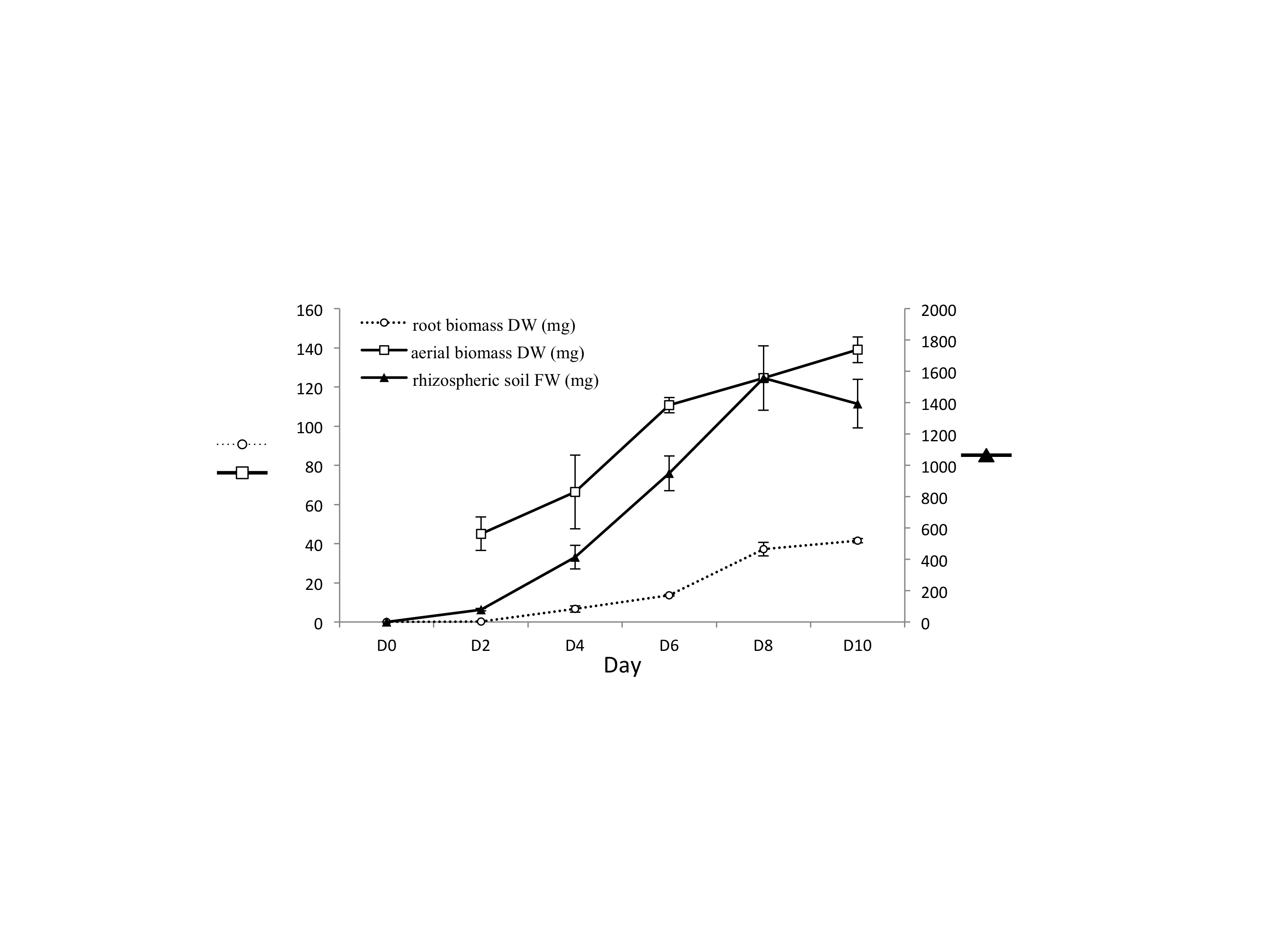

Supplement: Figure S1 — Evolution of root and aerial dry weight biomass, and rhizospheric soil fresh weight biomass in microcosms planted with ryegrass. Values are mean ± s.e.m. (n = 3). [file Image1.PNG]

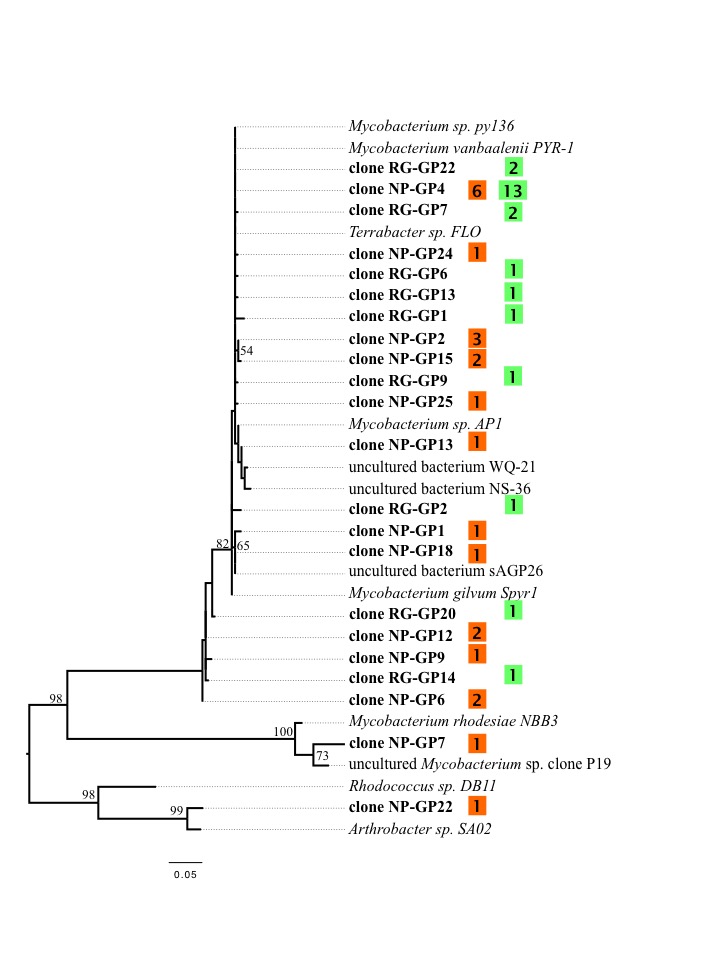

Supplement: Figure S2 — Maximum-likelihood (ML) phylogenetic reconstruction of PAH-RHD GP nucleotidic sequences cloned from the Neuves-Maison soil (in boldface), including publicly available sequences from reference strains and uncultured bacteria. The Tamura-Nei model was used for the ML analysis. Values of ML bootstrap support (100 resamplings) greater than 50% are reported. Unique sequences are represented by one selected clone; numbers of retrieved sequences from bare and bulk planted soil are shown in orange and green boxes, respectively. The tree was rooted on the Rhodococcus/Arthrobacter clade. The bar indicates 5% sequence divergence. [file Image2.JPEG]

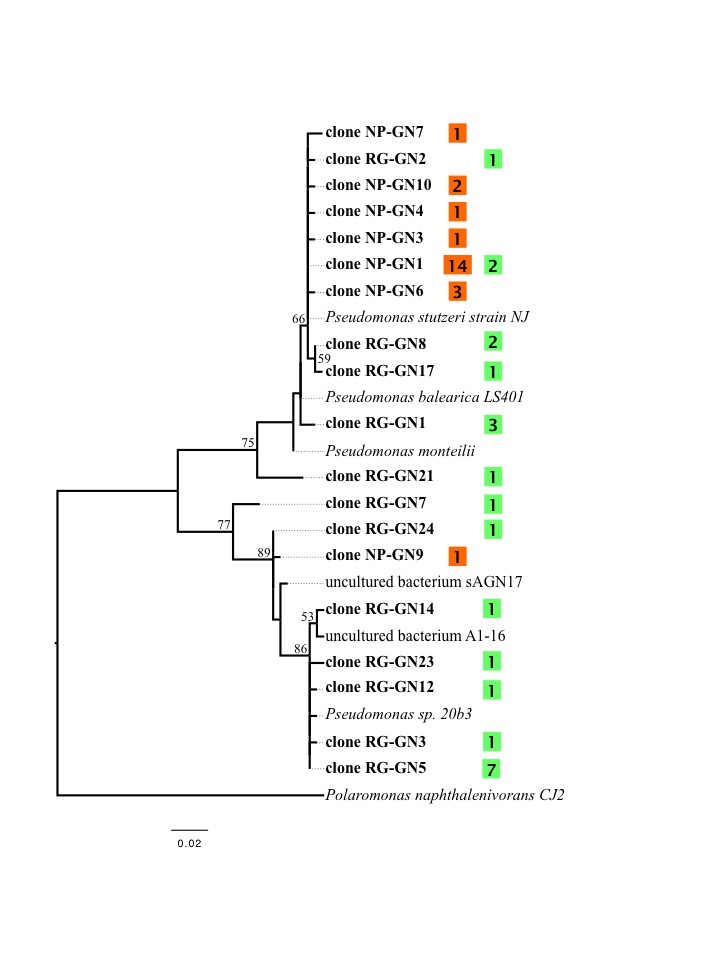

Supplement: Figure S3 — Maximum-likelihood (ML) phylogenetic reconstruction of PAH-RHD GN nucleotidic sequences cloned from the Neuves-Maison soil (in boldface), including publicly available sequences from reference strains and uncultured bacteria. The Tamura-Nei model was used for the ML analysis. Values of ML bootstrap support (100 resamplings) greater than 50% are reported. Unique sequences are represented by one selected clone; numbers of retrieved sequences from bare and bulk planted soil are shown in orange and green boxes, respectively. The tree was rooted on the Polaromonas branch. The bar indicates 2% sequence divergence. [file Image3.JPEG]

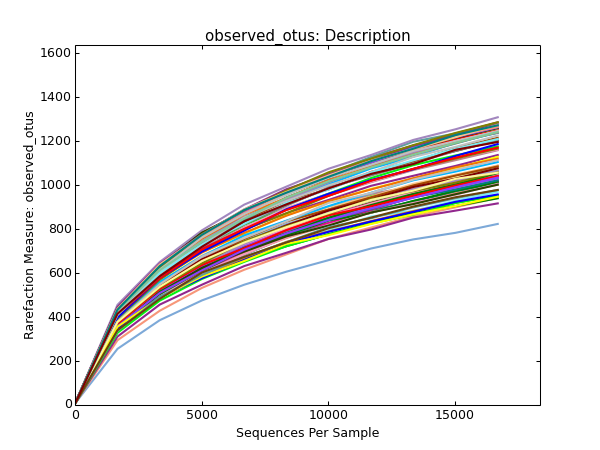

Supplement: Figure S4 — Rarefaction curves for OTUs clustered at 97% similarity. [file Image4.PNG]

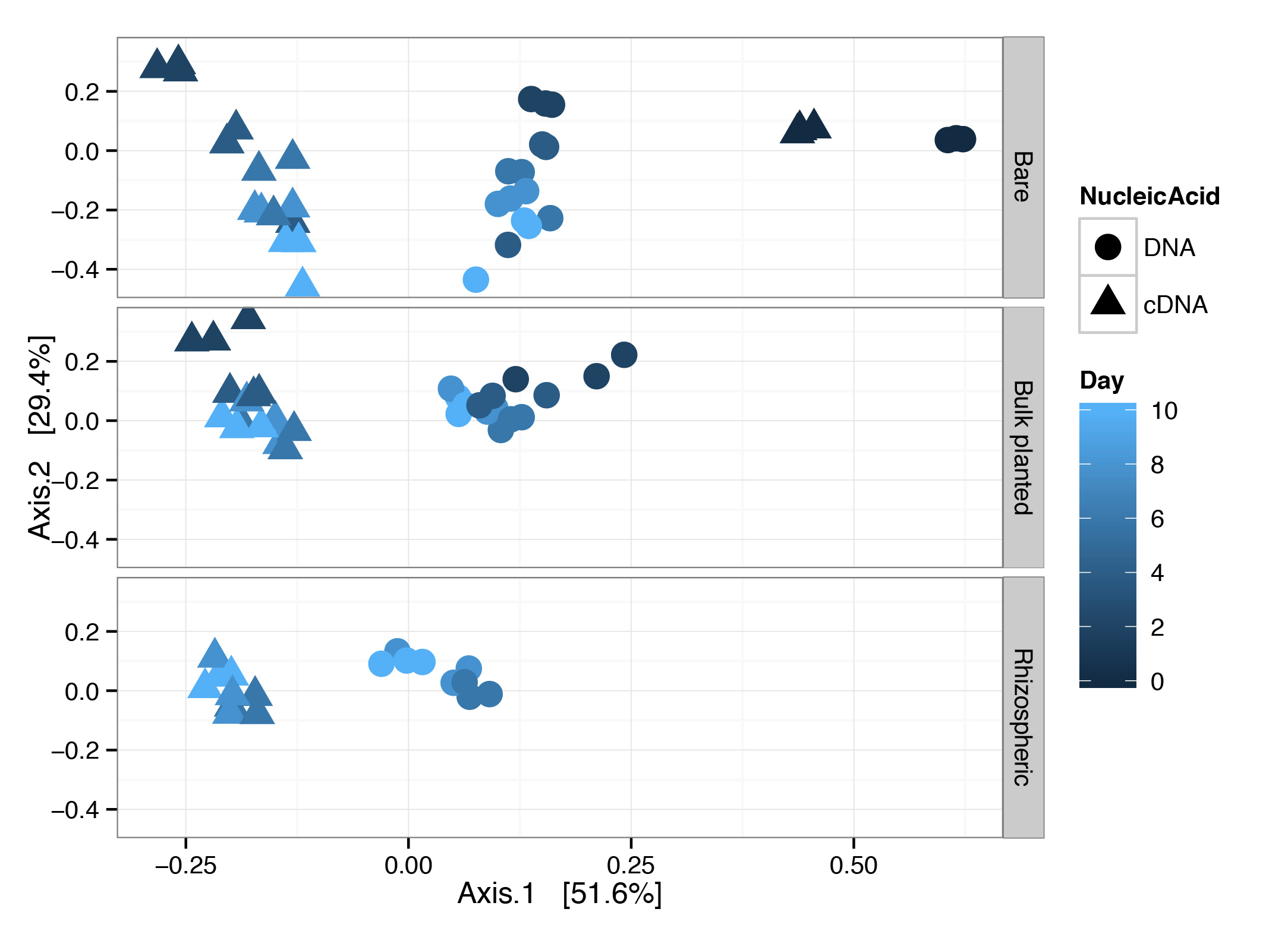

Supplement: Figure S5 — Principal Coordinates Analysis (PCoA) displaying the Morisita-Horn dissimilarity between soil bacterial communities, based on DNA and cDNA libraries. Data from bare, bulk planted and rhizospheric soil were analyzed all together but plotted on three superimposable panels for clarity. Permutational Analysis of Variance corroborated the effect of nucleic acid (DNA vs. cDNA; F = 43.0, P < 0.001, R2= 0.24) and type of samples (bare vs. bulk planted vs. rhizospheric; F = 11.8, P < 0.001, R2= 0.13) on the community composition, as well as an evolution with time (F = 33.7, P < 0.001, R2 = 0.19). [file Image5.JPEG]

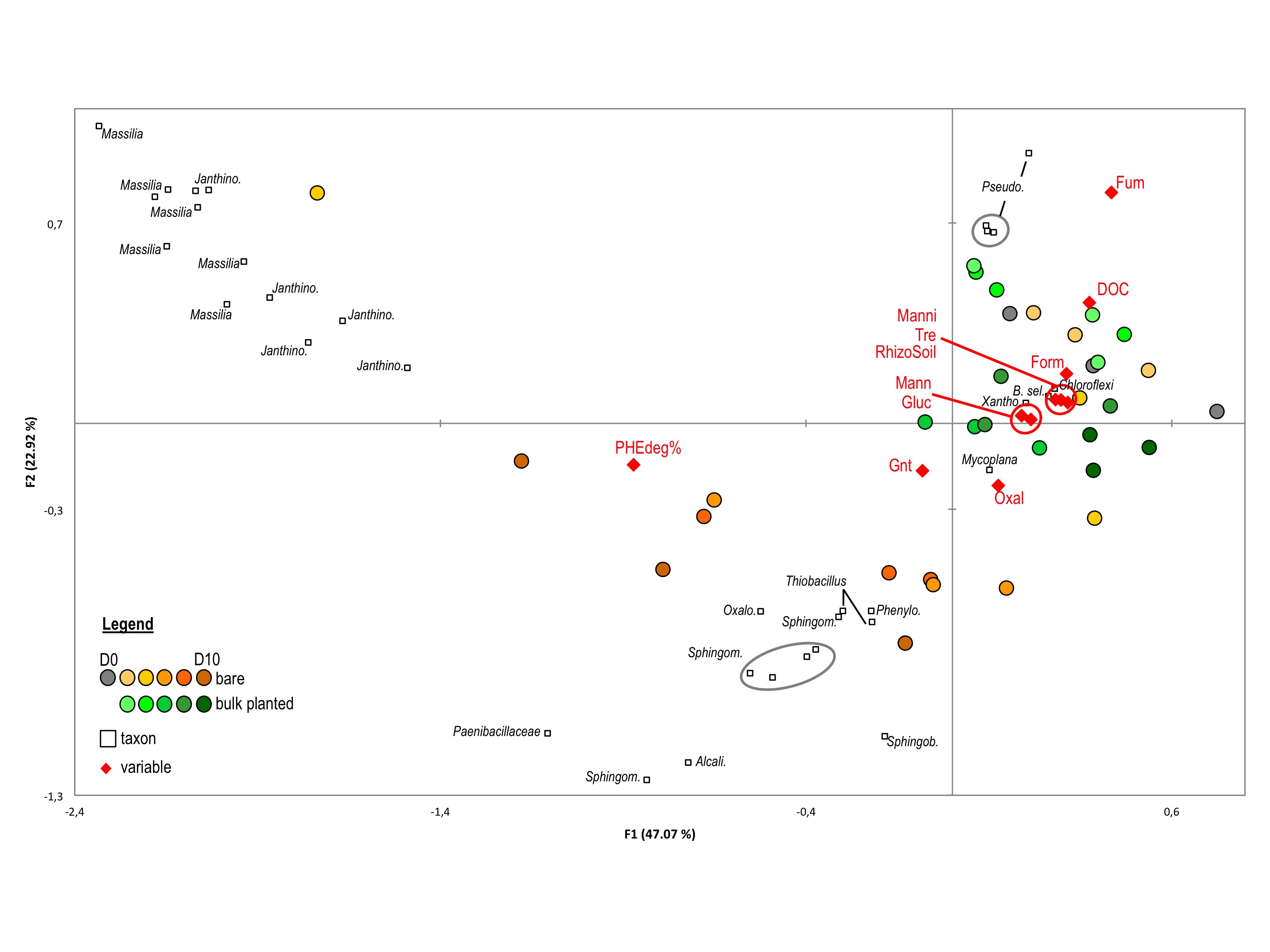

Supplement: Figure S6 — Canonical Correspondance Analysis based on the distribution of abundant OTUs (>1% in at least one sample) in DNA libraries. The model accounted for 59.9% of the total inertia, with a global P-value of 0.002. Circles represent samples from bare (green palette) or bulk planted soil (orange palette). Environmental variables (red diamonds) include percentage of PHE degradation (PHEdeg%), rhizospheric soil fresh weight (RhizoSoil), total dissolved organic carbon (DOC), glucose (Gluc), mannose (Mann), trehalose (Tre), mannitol (Manni), fumarate (Fum), oxalate (Oxal), gluconate (Gnt), and formate (Form). Squares represent OTUs with their taxonomic affiliation, abbreviated as follows: Janthino., Janthinobacterium; Sphingom., Sphingomonas; Alcali., unclassified Alcaligenaceae; Phenylo., Phenlyobacterium; Oxalo., unclassified Oxalobacteraceae; Sphingob., Sphingobium; Pseudo., Pseudomonas; B. sel., Bacillus selenatarsenatis; Xantho., unclassified Xanthomonadaceae. For clarity, only OTUs with contribution >1% to at least one axis were depicted. [file Image6.JPEG]
