# Supplementary material for: Short-Term Rhizosphere Effect on Available Carbon Sources, Phenanthrene Degradation, and Active Microbiome in an Aged-Contaminated Industrial Soil
Source: Front Microbiol. 2016 Feb 5;7:92. doi: 10.3389/fmicb.2016.00092 (PMC4742875; doi:10.3389/fmicb.2016.00092)
Supplement: Table S1 — Spearman correlation coefficient between DNA and cDNA read data. Differences between conditions were tested using ANOVA followed by post-hoc Tukey-Kramer test. Letters denote groups with significant differences (P < 0.05). [file Table1.docx]

**Table Supp 1:** Spearman correlation coefficient between DNA and cDNA read data. Differences between conditions were tested using ANOVA followed by post-hoc Tukey-Kramer test. Letters denote groups with significant differences (P<0.05).

|  | **Spearman's rho** | | |
| --- | --- | --- | --- |
|  | Bare | Bulk planted | Rhizospheric |
| Day 0 | 0.54 ± 0.01^bcd^ |  |  |
| Day 2 | 0.52 ± 0.00^d^ | 0.53 ± 0.00^cd^ |  |
| Day 4 | 0.56 ± 0.01^abc^ | 0.57 ± 0.00^abc^ |  |
| Day 6 | 0.57 ± 0.01^a^ | 0.58 ± 0.01^a^ | 0.59 ± 0.00^a^ |
| Day 8 | 0.56 ± 0.00^abc^ | 0.57 ± 0.01^ab^ | 0.57 ± 0.01^abc^ |
| Day 10 | 0.58 ± 0.01^a^ | 0.57 ± 0.00^ab^ | 0.56 ± 0.00^abc^ |
